# Tetrazolium Salt WST-8 as a Novel and Reliable Chromogenic Indicator for the Assessment of Boar Semen Quality

**DOI:** 10.3390/ani10122293

**Published:** 2020-12-04

**Authors:** Yu-Hsin Chen, Chean-Ping Wu, Hsiu-Lien Lin, Ren-Bao Liaw, Yung-Yu Lai, Ming-Che Wu, Lih-Ren Chen, Pei-Shiue Jason Tsai

**Affiliations:** 1 Physiology Division, Livestock Research Institute, Council of Agriculture, Tainan 71246, Taiwan; yhchen@mail.tlri.gov.tw (Y.-H.C.); hllin@mail.tlri.gov.tw (H.-L.L.); lrchen@mail.tlri.gov.tw (L.-R.C.); 2 Department of Animal Science, National Chiayi University, Chiayi 60004, Taiwan; wcp@mail.ncyu.edu.tw (C.-P.W.); liawrb@mail.tlri.gov.tw (R.-B.L.); 3 Livestock Management Division, Livestock Research Institute, Council of Agriculture, Tainan 71246, Taiwan; laiyy@mail.tlri.gov.tw (Y.-Y.L.); wumc@mail.tlri.gov.tw (M.-C.W.); 4 Institute of Biotechnology, National Chung Kung University, Tainan 70101, Taiwan; 5 Graduate Institute of Veterinary Medicine, National Taiwan University, Taipei 10617, Taiwan; 6 Research Center for Developmental Biology and Regenerative Medicine, National Taiwan University, Taipei 10617, Taiwan

**Keywords:** semen quality, boar, computer-assisted sperm analysis (CASA), flow cytometry, WST-8

## Abstract

**Simple Summary:**

Good semen quality is an essential factor for breeding success when artificial insemination is performed. However, semen quality can not fully be evaluated by merely sperm viability or motility. Standard semen quality evaluation requires costly automated computer-assisted sperm analysis or specific and laborious labeling procedures before particular parameters can be assessed by flow cytometry. In the current study, we examined whether the 2-[2-methoxy-4-nitrophenyl]-3-[4-nitrophenyl]-5-[2,4-disulfophenyl]-2H-tetrazolium (WST-8) assay, which is widely used in the cell biology field, can be applied to evaluate sperm viability, and moreover, whether the WST-8 reduction rate can correlate with multiple sperm parameters that are related to sperm quality. We demonstrated in this study that the WST-8 assay can be used as a rapid, affordable, and reliable method for the prediction of semen quality in boar.

**Abstract:**

A tetrazolium salt, 2-[2-methoxy-4-nitrophenyl]-3-[4-nitrophenyl]-5-[2,4-disulfophenyl]-2H-tetrazolium (WST-8), has been used widely to determine cell viability; however, its application in the field of reproduction is still limited due to this assay merely providing information regarding cell viability. The aim of this study was to correlate the WST-8 reduction rate with various sperm quality-related parameters (i.e., sperm viability, motility, progressive motility, acrosome integrity and mitochondria integrity) in order to provide a rapid, reliable and affordable assessment for boar semen quality evaluation. Using different ratios of active/damaged sperm cells, we first validated our sample preparations by standard flow cytometry and computer-assisted sperm analysis. Further analyses demonstrated that the most efficient experimental condition for obtaining a reliable prediction model was when sperm concentration reached 300 × 10^6^ cells/mL with the semen/cell-counting kit-8 (CCK-8^®^) ratio of 200/10 and incubated time of 20 min. Under this set up, the WST-8 reduction rate (differences on optic density reading value, ΔOD at 450 nm) and sperm parameters were highly correlated (*p* < 0.01) for all sperm parameters evaluated. In the case of limited semen samples, a minimal semen concentration at 150 × 10^6^ cells/mL with the semen/CCK-8^®^ ratio of 200/20 and incubation time for 30 min could still provide reliable prediction of sperm parameters using the WST-8 assay. Our data provide strong evidence for the first time that the WST-8 assay could be used to evaluate boar semen quality with great potential to be applied to different mammalian species.

## 1. Introduction

Assisted reproductive technology (ART) including artificial insemination (AI) has been utilized by the swine industry to improve reproductive performance for more than 50 years [1]. Due to breed variations and post-ejaculation semen handling procedures, comprehensive and precise evaluation of sperm quality before AI is essential to correlate boar fertilizing potential and to increase fertilization success [2,3,4,5]. Spermatozoon is a specialized type of cell with polarized domains, including the sperm head, mid-piece, and tail. The sperm head, in addition to chromatin, also contains a large Golgi-derived acrosome with various hydrolytic enzymes that are essential for sperm-zona pellucida penetration [6]. The sperm mid-piece on the other hand is packed with mitochondria that are critical for sperm motility [7]. Functional integrity of all the above-mentioned compartments is essential for successful fertilization.

Various techniques and parameters have been developed and used to access functional integrity of sperm cells [3,8]. However, for the purpose of routine and practical applications, semen and sperm evaluation methods must be rapid, accurate, affordable and with high reproducibility. Computer-assisted sperm analysis (CASA) is one of the most commonly used technologies to assess sperm quality since the late 1980s [9,10,11]. Analytical information derived from CASA allows sperm images and motility-related parameters to be captured and analyzed by specific software with defined criteria. This routinely used automated system mainly measures sperm concentration and sperm motility-based factors. Although sperm motility is one of the basic parameters providing diagnostic and prognostic information for clinical gynecologists or in vitro fertilization (IVF) operators and valuable and quick indications on the selection of “healthy sperm”, it provides limited information on other intrinsic but critical sperm parameters (e.g., DNA or chromosomal abnormality, sperm membrane integrity etc.). Another widely used technique for evaluating sperm quality is flow cytometry (FC) in combination with various fluorescence probes. FC can be utilized to detect specific damages to sperm and to evaluate the severity of insults by calculating the proportional numbers of sperm cells that are labeled with specific fluorescent molecules [12,13,14,15]. Objective evaluation and individual fertility prediction might be possible by introducing automated flow cytometric semen analysis with different vital stains to distinguish normal or defective sperm phenotypes [5]. Recently developed multicolor flow cytometric analysis, including calcein violet, propidium iodide, phycoerythrin-conjugated lectin of Arachis hypogea, Fluo-4, and cyanine dye DiIC_1_ (5), was configured by a three-laser flow cytometer, and enables simultaneous assessment on sperm esterase activity, plasma membrane integrity, acrosomal status, intracellular Ca^2+^ levels, and mitochondrial membrane potential, respectively [12]. Despite both CASA and FC providing accurate, fast, and large-scale semen evaluation outcomes, both approaches are relatively expensive and complex pieces of equipment are required and may not be affordable for every laboratory.

The 3(4,5-dimethylthiazol-2-y1)-2,5-diphenyltetrazolium bromide (MTT) assay has been used extensively for assessing the viability of cells, including in sperm [16,17,18,19]. When sperm cells are viable, MTT is reduced by nicotinamide adenine dinucleotide (NADH) within functional mitochondria, forming crystallized water-insoluble formazan. However, organic solvent is required to solubilize the purple-colored crystal, of which the procedure itself often leads to inconsistent results when handled improperly. In contrast, 2-[2-methoxy-4-nitrophenyl]-3-[4-nitrophenyl]-5-[2,4-disulfophenyl]-2H-tetrazolium (WST-8) is a highly water-soluble disulfonated tetrazolium salt that forms water-soluble chromogenic formazan upon assessment; therefore, it has been used to evaluate cell viability with minimized potential for errors [16,20,21,22]. Based on the above-mentioned properties, the WST-8 assay has been considered as a more reliable method to estimate cell viability compared to the MTT assay. Although the WST-8 assay is a facile, inexpensive and reliable method to assess cell viability, to the best of our knowledge, besides a single report in cockerel [22], the WST-8 assay has never been applied to assess sperm viability in boar or other mammals. Therefore, the aim of the current study is to establish a standard protocol and working procedure for the WST-8 assay for boar semen quality assessment, and to correlate the WST-8 reduction rate with various sperm quality-related parameters (i.e., sperm viability, motility, progressive motility, acrosome integrity and mitochondria integrity) measured from flow cytometry and CASA, in order to provide a rapid, reliable and affordable assessment for boar semen quality evaluation.

## 2. Methods and Materials

### 2.1. Semen Collection and Preparation for the WST-8 Assay

All experiments were carried out in accordance with the regulations regarding ethical treatments in relation to animal use. Animal handling and materials used were approved by the institutional animal care and use committee at Livestock Research Institute (LRI IACUC, no. 105-10), and under the surveillance and guidance of a certified veterinarian. Four Duroc–Meishan crossbred boars of 18–26 months of age were used in this study. For each individual boar, at least 3 ejaculates were collected during routine daily practice. Sperm concentration was determined immediately by an automated photometer (SpermCue, Minitube, Tiefenbach, Germany). In order to establish standard curves and correlate the relationships between the WST-8 reduction rate and sperm viability, sperm motility, acrosome and mitochondria integrity, a modified methodology was developed based on Lin et al. [22]. Briefly, fresh semen samples were randomly divided into active (samples with alive sperm cells) and compromised (negative control, samples with damaged sperm cells) groups. Semen samples of the active group with alive sperm cells were kept at room temperature (RT) before assessments. To structurally and functionally compromise sperm integrity, sperm cells were plunged into liquid nitrogen and thawed at 37 °C for two cycles. To build up standard curves, semen samples were prepared by mixing active/compromised semen samples at a proportion of 0/10, 2/8, 4/6, 6/4, 8/2, and 10/0 (*v*/*v*, in a total 200 μL), respectively. To establish the best sperm concentration range that is suitable for the WST-8 assay, the above-mentioned semen mixtures were subsequently diluted with pre-warmed (37 °C) phosphate buffered saline (PBS) (Lonza, Basel, Switzerland) to obtain a sperm concentration ranging from 300, 150, 75, to 37.5 × 10^6^ sperm cells/mL. A time-dependent analysis from 0 to 60 min with a 10 min time interval was also performed to elucidate the reduction activity of WST-8 in mammalian sperm samples. Moreover, to correlate sperm factors with the WST-8 reduction rate, sperm viability, acrosome integrity, mitochondria function and sperm motility were analyzed with FC, and CASA, respectively, and the same semen preparation was parallelly subjected to WST-8 evaluation to obtain an optical density (O.D.) value for later correlation analysis.

### 2.2. WST-8 Assay

Commercially available cell counting kit-8 (CCK-8^®^) (Bimake, Houston, TX, USA) was used for the WST-8 assay according to the manufacturer’s instructions. CCK-8^®^ is a chromogenic redox indicator that utilizes a tetrazolium salt, WST-8, to form an orange color and water-soluble formazan upon the reduction by cellular dehydrogenases [20]. Similar to MTT, the intensity of the color is proportional to the number of alive cells [20,21]. For each sample, 3 technical repeats of 100 μL semen samples were applied in a 96-well microplate (Greiner Bio-One, Kremsmünster, Austria). Then, 10 μL CCK-8^®^ solution was subsequently added into each well, sample mixtures were subjected to a spectrophotometer (BioTek, Winooski, VT, USA), and the absorbance was recorded at a wavelength of 450 nm after incubation at 37 °C for 10, 20, 30, 40, 50, and 60 min, respectively. To determine the efficiency and the reaction limitation of the WST-8 assay, and to obtain the most optimal ratio between the semen and CCK-8^®^ solution upon the WST-8 assay, 200 μL semen samples were also mixed with 10 or 20 μL CCK-8^®^ solution.

### 2.3. Sperm Functional Integrity Analyses by Flow Cytometry

To evaluate different sperm parameters that are relevant for sperm structural and functional integrity, Guava^®^ EasyCyte micro-capillary flow cytometry (Guava Technologies Inc., Hayward, CA, USA) was applied. For sperm viability analysis, the EasyKit^TM^ Viability kit (cat.# 024708, IMV Technologies, L’Aigle, France; https://www.imv-technologies.com/documents/EASYKIT-1-FY-ENG.pdf) containing SyBr14 and propidium iodide (PI) stains with differential permeability to alive (membrane intact) and dead (membrane damaged) cells was used according to the manufacturer’s instructions. Sperm–reagent mixtures were incubated at 37 °C for 10 min before evaluation with Guava^®^ EasyCyte. A total number of 5000 cells were analyzed for each sample, and results are expressed as a percentage. Sperm acrosome integrity was evaluated by EasyKit^TM^ acrosome integrity (cat.# 025293, IMV Technologies), which contains peanut agglutinin (PNA), cell-permeant orange fluorescent nucleic acid stain, Syto83^TM^ and PI dyes to simultaneously assess disrupted acrosomes within live or dead sperm. Sperm–reagent mixtures were allowed to react at 37 °C for 45 min in the dark before the signal was read with the Guava^®^ EasyCyte. Sperm mitochondria activity and functional integrity were measured with EasyKit^TM^ mitochondrial activity (cat.# 024864, IMV Technologies). The kit contains JC-1 dye, which is known to differentiate polarized and depolarized mitochondria with an apparent orange/red or green color, respectively. Sperm–reagent mixtures were incubated at 37 °C for 30 min in the dark and analyzed with Guava^®^ EasyCyte. Five thousand events were counted, and the results are displayed as the percentage of polarized mitochondria.

### 2.4. Computer-Assisted Sperm Analysis for Motility-Related Sperm Parameters

Sperm motility was assessed with a CEROS II™ CASA device (Hamilton Thorne Inc., Beverly, MA, USA). Sperm concentration was adjusted to 30 × 10^6^ cells/mL using pre-warmed (37 °C) PBS, and a 2.5 μL aliquot was used and analyzed with a four-chamber CellVision counting slide (CellVision Technologies, Heerhugowaard, The Netherlands). Analysis was conducted on a temperature-controlled stage at 37 °C as suggested by the company. Definition and threshold values for all sperm parameters followed default instructions from the company. Five field images were captured for each sample, the image capture was set to 60 frames/s, a total of 45 frames were recorded per examination field. By the use of an automated stage, a total of 225 frames were taken per experimental sample, and results were further analyzed and are expressed in percentage for each parameter.

### 2.5. Statistical Analysis

All analyses were performed using SAS software (version 9.3, SAS Institute Inc., Cary, NC, USA). The sperm membrane integrity (%), acrosomal status (%), mitochondrial activity (%), total motility (%) and progressive sperm (%) were analyzed using one-way ANOVA. Differences in the means were analyzed by Tukey–Kramer’s honestly significant difference (HSD) test. Values are expressed as mean ± SEM. The correlation coefficient (CORR) procedure was applied to calculate the Pearson correlation coefficient (r) between the WST-8 reduction rate and the sperm parameters of boar semen. *p* value < 0.01 was considered as significant. Hereafter, data were analyzed by the regression (REG) procedure of SAS™, and a simple linear model was used to obtain the standard curves for the prediction of sperm viability, acrosome integrity, mitochondria activity, total motility, and progressive sperm.

## 3. Results

### 3.1. Flow Cytometry and CASA Analyses Showed Consistent and Positive Correlations among Different Sperm Sample Preparations

After semen ejaculates were obtained, general characteristics of the ejaculates were immediately evaluated with CASA. On average (from 12 ejaculates), 88.80 ± 8.80 mL of semen volume with a sperm concentration of 5.30 ± 0.70 × 10^8^ sperm/mL, 81.4 ± 5.9% motility and 40.80 ± 5.00% progressive motility was obtained before further manipulation for experimental conditions. Boar sperm parameters evaluated by standard flow cytometry and CASA approaches are presented in Table 1. The percentages of viability, acrosome integrity, mitochondrial activity, motile sperm, and progressive sperm of 0/10, 2/8, 4/6, 6/4, 8/2 and 10/0 active/compromised sperm samples are shown in Table 1. More active sperm cells result in higher percentages of viability, motile sperm, progressive, acrosome and mitochondria intact ratios. Our results in Table 1 demonstrate that the semen samples prepared for standardization are consistent between the two independent approaches (i.e., flow cytometry and CASA). Moreover, values measured in both methods were also consistent with the ratio of active/compromised sperm cells in different sample preparations.

### 3.2. Positive Correlation between the WST-8 Reduction Rate and Boar Sperm Parameters

Pearson’s correlation coefficients between the WST-8 reduction rate (differences on optic density reading value, ΔOD at 450 nm) and various boar sperm parameters at different sperm concentrations and incubation time points were measured and calculated accordingly. As shown in Table 2, a sperm concentration- and incubation time-dependent increase in positive correlation between the WST-8 reduction rate and sperm parameters evaluated was observed in all groups when a standard semen/CCK-8^®^ solution mixture of 100/10 (μL, *v*/*v*) was used (Table 2). When the sperm concentration reached 300 × 10^6^ cells/mL, the WST-8 reduction rate was highly correlated (*r* ≥ 0.78, *p* < 0.01) with all sperm parameters after 20 min incubation time, suggesting that a sperm concentration of 300 × 10^6^ cells/mL and a minimum 20 min incubation time are required for a reliable prediction of sperm parameters using the WST-8 assay. We next tested whether different ratios between semen volume and CCK-8^®^ solution can result in the improvement of the assay in terms of the amount of CCK-8^®^ solution used or incubation time needed. We demonstrated in Table 3 that when sperm concentration was at 300 × 10^6^ cells/mL, the WST-8 reduction rate and different boar sperm parameters were highly correlated (*r* ≥ 0.78, *p* < 0.01), even after 10 min when the semen/CCK-8^®^ ratio was modified to 200/20 or 200/10. Surprisingly, when the sperm concentration was at 150 × 10^6^ cells/mL, highly positive correlations (*r* ≥ 0.88, *p* < 0.01) between the WST-8 reduction rate and boar sperm parameters were also calculated after 30 min incubation when the semen/CCK-8^®^ ratio was 200/20, indicating that a sufficient amount of reacting semen/reagent mixture or a prolonged incubation time can also achieve reliable prediction (Table 3).

### 3.3. Models and Formula for Interpolation of Boar Semen Quality Using the WST-8 Assay

**Figure 1:**
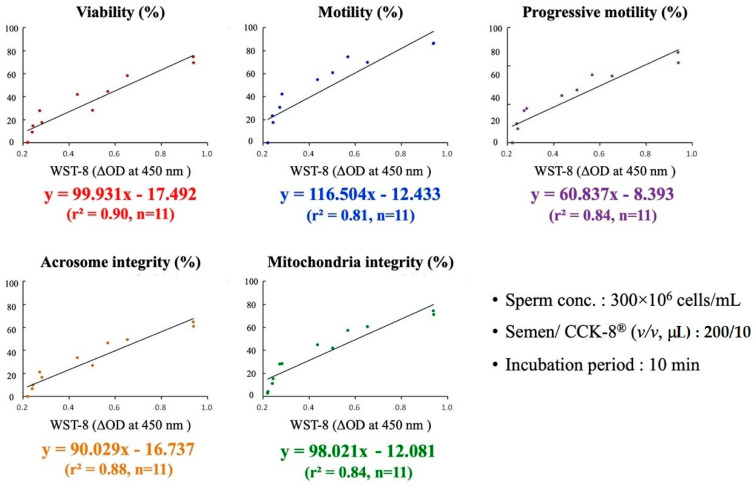
Linear regression and prediction models for sperm concentration of 300 × 10^6^ cells/mL. Prediction model and formula between the WST-8 reduction rate (ΔOD) and boar sperm viability (%), sperm motility (%), progressive sperm (%), acrosome integrity (%) and mitochondrial integrity (%), at a sperm concentration of 300 × 10^6^ cells/mL after 10 min incubation at 37 °C with a semen/CCK-8^®^ ratio of 200/10.

**Figure 2:**
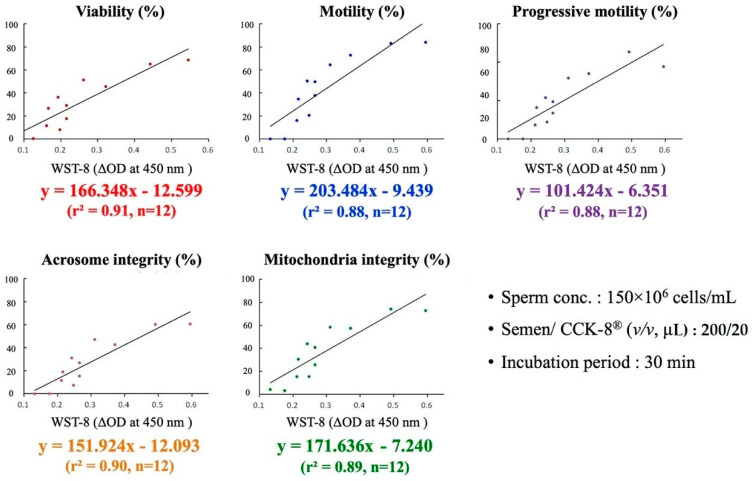
Linear regression and prediction models for sperm concentration of 150 × 10^6^ cells/mL. Prediction model and formula between the WST-8 reduction rate (ΔOD) and boar sperm viability (%), sperm motility (%), progressive sperm (%), acrosome integrity (%) and mitochondrial integrity (%), at a sperm concentration of 150 × 10^6^ cells/mL after 30 min incubation at 37 °C with a semen/CCK-8^®^ ratio of 200/20.

Based on the correlation coefficient results presented in Table 2 and Table 3, linear regression analysis for the prediction of boar sperm quality using the WST-8 assay was performed separately under the most optimal semen/CCK-8^®^ combination protocol (in terms of short incubation time and minimal reagents used) for sperm concentrations of 300 × 10^6^ cells/mL and 150 × 10^6^ cells/mL. As shown in Figure 1, when the sperm concentration was 300 × 10^6^ cells/mL with the semen/CCK-8^®^ ratio of 200/10 for 20 min incubation, the prediction formula for sperm viability was (%) = 99.93x − 17.49; sperm motility (%) = 116.50x − 12.43; progressive motility (%) = 60.84x − 8.39; acrosome integrity (%) = 90.03x − 16.74; and mitochondrial integrity (%) = 98.02x − 12.08, with WST-8 OD value at 450 nm as “x” (Figure 1). On the other hand, when the sperm concentration was 150 × 10^6^ cells/mL with the semen/CCK-8^®^ ratio of 200/20 and incubated for 30 min, the prediction formula for sperm viability was (%) = 166.35x − 12.60; motile sperm (%) = 203.48x − 9.44; progressive sperm (%) = 101.42x − 6.35; acrosome integrity (%) = 151.94x − 12.09; and mitochondrial activity (%) = 171.64x − 7.24 with WST-8 OD value at 450 nm as “x” (Figure 2).

## 4. Discussion

WST-8-based analysis, a colorimetric proliferation assay, has been extensively used to determine the viability of cells [16,23,24]. However, besides a recent report that stated its usage on the determination of avian sperm viability [22], no other information is available regarding its application on mammalian spermatozoa. In this study, we demonstrate for the first time that the WST-8 assay could be used as a reliable chromogenic indicator for semen quality in mammals. Our study shows that the WST-8 reduction rate is highly correlated to boar sperm parameters, including sperm viability, motility, progressive motility, acrosome integrity and mitochondria functionality. As WST-8 has already been formulated in many commercialized ready-to-use solution kits, our study provides evidence supporting the use of WST-8 as a simple, rapid and reliable measurement for practical applications when evaluating semen quality in boar.

Semen quality is mostly assessed by the percentage of viable and motile sperm cells present in the semen using cell viability assay (e.g., MTT) [25], flow cytometry [13,26] and automated computer-assisted sperm analysis [27]); however, these analyses exhibit either considerable variations between operators or require costly and laborious support from the laboratory. van den Berg and Byun et al. used the MTT assay to evaluate boar sperm viability and showed that the correlation coefficients between MTT and sperm viability evaluated by eosin-nigrosin staining were highly correlated after 1–4 h co-incubation time [28,29]. When compared with these studies, our results indicate that with the WST-8 protocol developed in the current study, the incubation period for the semen/CCK-8^®^ mixture could be reduced to as short as 10 min by increasing the sperm number to 300 × 10^6^ cell/mL. This significant improvement is likely due to different sensitivities and reaction rates between MTT and the WST-8 assay as the conversion of 3-(4,5-dimethylthiazol-2-yl)-5-(3-carboxymethoxyphenyl)-2-(4-sulfophenyl)-2H-tetrazolium (MTS) tetrazolium salt (WST-8) into water-soluble formazan product by NADH is faster than converting 3-(4,5-dimethylthiazol-2-yl)-2,5-diphenyltetrazolium bromide (MTT) into its water-insoluble formazan [23,30,31]. On the other hand, despite the fact that flow cytometry can be used to evaluate specific sperm molecules on a relatively large scale [12,32], flow cytometry-based sperm analysis requires specific labeling of designed molecules, and the number of samples that can be evaluated is limited within a period of time [13].

In the current study, a positive correlation between the WST-8 reduction rate and semen parameters was observed. Mitochondria are important cellular organelles that provide an energy source for sperm motility; thus, mitochondria health reflected on the WST-8 reduction rate can therefore correlate to sperm viability and motility-related parameters. It is of particular interest that we observed a positive correlation between the WST-8 reduction rate and acrosome integrity, since no mitochondria or NADH have been reported in the sperm acrosome. The high correlation is likely due to the acrosome intact sperm mostly having a quiescent status, and under this condition, sperm mitochondria are at a polarized condition. Therefore, the positive correlation observed in the current study between the WST-8 reduction rate and acrosome integrity might be an indirect connection rather than a direct association between the WST-8 reduction rate and acrosome status per se.

In this study, we showed that the correlation coefficients between the WST-8 reduction rate and boar sperm parameters were highly and positively correlated (*r* = 0.78–0.90) when the semen concentration reached 300 × 10^6^ cells/mL. Moreover, from the economic and experimental efficiency (in terms of time and reagents used) point of view, semen/CCK-8^®^ ratio of 200/10 (*v*/*v*) is considered as the most optimal combination that can be used to obtain a reliable prediction model within a minimal experimental time frame. As showed in Table 3, our data also indicated that for the WST-8 assay, to obtain a reliable measurement, a balance between the amount of reagent (WST-8) and the number of functional mitochondria is required. Based on our data, a reliable correlation between WST-8 and sperm parameters requires a minimal amount of colorimetric formazan to be formed and measured by ELISA reader. Thus, depending on the constant changing conditions in the field at each institute, the choice of sperm concentration, incubation time, and semen/CCK-8 ratio can be varied. Nevertheless, we also showed that in the case of limited and valuable semen samples from endangered or wildlife species (e.g., pangolin, black bear, great apes) [19,33] or from species where it is difficult to obtain a large quantity of the semen (e.g., rodent species), a minimal sperm concentration of 150 × 10^6^ sperm cells/mL with semen/CCK-8^®^ ratio of 200/20 and incubation time for 30 min will allow a reliable evaluation of boar semen quality using the WST-8 assay. Therefore, our study provides the first evidence that the WST-8 assay is a reliable chromogenic indicator for semen quality evaluation in boar. Although sperm morphology differs between mammals, we believe that the WST-8-based colorimetric detection of NADH for quantitative dehydrogenase analysis could be applied widely in semen quality evaluation among different mammalian species as the dehydrogenase activity shares a high degree of similarity within mitochondria among different mammalian species. However, as animals of different species may have species-specific variations upon using this assay, validation (by either flow cytometry or CASA) and careful interpretation of the data are highly suggested before applications on other animal species other than boar are undertaken.

## 5. Conclusions

In conclusion, our study demonstrated that the WST-8 assay, a rapid and affordable assay, provides simultaneous estimation on many sperm quality-related factors with high reproducibility and reliability based on the WST-8 reduction rate. All these advantages make the WST-8 assay a simple and practical tool for evaluating boar semen quality in a simply equipped lab or a pig breeding farm.

## Figures and Tables

**Table 1:** Boar sperm parameters assessed with flow cytometry (FC) and computer-assisted sperm analysis (CASA).

Active/Compromised Sperm Ratio	Flow Cytometry Assessment			CASA Assessment	
	Viability (%)	Acrosome Integrity (%)	Mitochondrial Integrity (%)	Motile Sperm (%)	Progressive Sperm (%)
0/10	0.40 ± 0.11 ^a^	0.23 ± 0.18 ^a^	5.50 ± 1.87 ^a^	0.00 ± 0.00 ^a^	0.00 ± 0.00 ^a^
2/8	12.47 ± 1.32 ^b^	9.88 ± 1.17 ^b^	16.62 ± 2.30 ^b^	18.95 ± 1.50 ^b^	8.18 ± 0.52 ^b^
4/6	24.03 ± 2.21 ^c^	18.83 ± 0.98 ^c^	29.86 ± 1.83 ^c^	35.35 ± 1.92 ^c^	16.17 ± 0.64 ^c^
6/4	36.50 ± 3.03 ^d^	30.10 ± 1.31 ^d^	44.60 ± 1.75 ^d^	52.05 ± 2.39 ^d^	23.72 ± 1.33 ^d^
8/2	52.33 ± 2.73 ^e^	46.03 ± 1.56 ^e^	58.78 ± 0.65 ^e^	67.37 ± 2.63 ^e^	32.92 ± 1.18 ^e^
10/0	72.05 ± 3.06 ^f^	62.65 ± 1.47 ^f^	73.80 ± 0.95 ^f^	81.37 ± 2.42 ^f^	40.75 ± 2.02 ^f^

Values are expressed as means ± SEM from 36 samples in 6 independent experiments. ^a–f^ indicates significant differences (*p* < 0.01) between different active/compromised ratios within the same column of a tested parameter. Statistical analysis was performed using one-way ANOVA.

**Table 2:** Pearson’s correlation coefficients between the 2-[2-methoxy-4-nitrophenyl]-3-[4-nitrophenyl]-5-[2,4-disulfophenyl]-2H-tetrazolium (WST-8) reduction rate and boar sperm parameters in different sperm concentrations and incubation times.

Parameters	Correlation Coefficients (r)			
	Sperm Concentration (10^6^ cells/mL)			
	300	150	75	37.5
10 min				
Viability (%)	0.48	−0.61	−0.18	−0.16
Acrosome integrity (%)	0.54	−0.49	−0.31	−0.16
Mitochondrial integrity (%)	0.30	−0.86 *	−0.62	−0.40
Motile sperm (%)	0.52	−0.46	−0.23	−0.15
Progressive sperm (%)	0.51	−0.45	−0.22	−0.16
20 min				
Viability (%)	0.81 *	−0.60	−0.06	−0.06
Acrosome integrity (%)	0.88 *	−0.49	−0.20	−0.12
Mitochondrial integrity (%)	0.81 *	−0.90 *	−0.58	0.41
Motile sperm (%)	0.81 *	−0.48	−0.11	−0.11
Progressive sperm (%)	0.78 *	−0.49	−0.10	−0.10
30 min				
Viability (%)	0.87 *	−0.28	0.10	−0.10
Acrosome integrity (%)	0.92 *	−0.19	−0.04	−0.08
Mitochondrial integrity (%)	0.85 *	−0.88 *	0.42	0.23
Motile sperm (%)	0.84 *	−0.20	0.08	−0.04
Progressive sperm (%)	0.82 *	−0.21	0.09	−0.03
40 min				
Viability (%)	0.89 *	−0.04	0.32	0.08
Acrosome integrity (%)	0.91 *	0.05	0.30	0.09
Mitochondrial integrity (%)	0.85 *	−0.89 *	0.53	0.45
Motile sperm (%)	0.82 *	0.03	0.45	0.12
Progressive sperm (%)	0.82 *	0.02	0.45	0.12
50 min				
Viability (%)	0.89 *	0.36	0.34	0.39
Acrosome integrity (%)	0.89 *	0.42	0.42	0.56
Mitochondrial integrity (%)	0.85 *	−0.47	0.34	0.60
Motile sperm (%)	0.80 *	0.36	0.50	0.59
Progressive sperm (%)	0.81 *	0.36	0.49	0.60
60 min				
Viability (%)	0.88 *	0.51	0.61	0.75 *
Acrosome integrity (%)	0.87 *	0.56	0.68	0.78 *
Mitochondrial integrity (%)	0.84 *	−0.09	**0.85** *	**0.72**
Motile sperm (%)	0.78 *	0.50	0.74 *	0.84 *
Progressive sperm (%)	0.79 *	0.50	0.74 *	0.85 *

WST-8 reduction rate = ΔOD at 450 nm. A standard semen/cell-counting kit-8 (CCK-8^®^) solution mixture of 100/10 (μL, *v*/*v*) was used. Data presented are the average values collected from 36 samples from 6 independent experiments, for each value, 6 measurements (*n* = 6) were performed. Asterisks (*) indicate that the correlation coefficient value differs significantly (*p* < 0.01) from zero. Bold font represents positive correlation.

**Table 3:** Pearson’s correlation coefficients between the WST-8 reduction rate and boar sperm parameters in different ratios of semen/cell-counting kit-8 (CCK-8^®^) solution mixtures (*v*/*v*).

	Correlation Coefficients (r)			
Sperm Concentration (10^6^ cells/mL)	300		150	
Semen/CCK-8^®^ (*v*/*v*, μL)	200/20	200/10	200/20	200/10
10 min				
Viability (%)	0.88 *	0.90 *	0.42	−0.14
Acrosome integrity (%)	0.87 *	0.88 *	0.41	−0.12
Mitochondrial activity (%)	0.83 *	0.84 *	0.44	−0.09
Motile sperm (%)	0.78 *	0.81 *	0.49	0.01
Progressive sperm (%)	0.81 *	0.84 *	0.44	−0.12
20 min				
Viability (%)	0.93 *	0.95 *	0.66	0.22
Acrosome integrity (%)	0.96 *	0.95 *	0.65	0.23
Mitochondrial activity (%)	0.95 *	0.94 *	0.66	0.24
Motile sperm (%)	0.92 *	0.91 *	0.68	0.31
Progressive sperm (%)	0.94 *	0.93 *	0.68	0.22
30 min				
Viability (%)	0.94 *	0.94 *	0.91 *	0.69
Acrosome integrity (%)	0.97 *	0.96 *	0.90 *	0.70
Mitochondrial activity (%)	0.97 *	0.95 *	0.89 *	0.69
Motile sperm (%)	0.96 *	0.93 *	0.88 *	0.70
Progressive sperm (%)	0.966 *	0.945 *	0.881 *	0.684
40 min				
Viability (%)	1.00 *	1.00 *	0.89 *	0.81 *
Acrosome integrity (%)	1.00 *	1.00 *	0.89 *	0.80 *
Mitochondrial activity (%)	1.00 *	1.00 *	0.87 *	0.78 *
Motile sperm (%)	1.00 *	1.00 *	0.85 *	0.78 *
Progressive sperm (%)	1.00 *	1.00 *	0.86 *	0.77 *
50 min				
Viability (%)	1.00 *	1.00 *	0.90 *	0.82 *
Acrosome integrity (%)	1.00 *	1.00 *	0.91 *	0.82 *
Mitochondrial activity (%)	1.00 *	1.00 *	0.88 *	0.79 *
Motile sperm (%)	1.00 *	1.00 *	0.87 *	0.77 *
Progressive sperm (%)	1.00 *	1.00 *	0.89 *	0.78 *
60 min				
Viability (%)	1.00 *	1.00 *	0.91 *	0.83 *
Acrosome integrity (%)	1.00 *	1.00 *	0.92 *	0.83 *
Mitochondrial activity (%)	1.00 *	1.00 *	0.90 *	0.81 *
Motile sperm (%)	1.00 *	1.00 *	0.88 *	0.79 *
Progressive sperm (%)	1.00 *	1.00 *	0.91 *	0.80 *

WST-8 reduction rate = ΔOD at 450 nm. Data presented are the average values collected from 36 samples from 6 independent experiments, for each value, 6 measurements (*n* = 6) were performed. Asterisks and bold font (*) indicate that the positive correlation coefficient value differs significantly (*p* < 0.01) from zero.
